# Impact of Coulomb Correlations on Magnetic Anisotropy in Mn_3_Ga Ferrimagnet

**DOI:** 10.1038/s41598-017-13276-5

**Published:** 2017-10-16

**Authors:** Srijan Kumar Saha, Zhen Liu, Gargi Dutta

**Affiliations:** 1 0103, Khaihata, Malda, 732142 West Bengal India; 2 Department of Physics, Beijing Normal University, Beijing, 100875 China; 3 Department of Physics, Balurghat College, Balurghat, 733101 West Bengal India

## Abstract

Traditional density functional theory (DFT) miserably fails to reproduce the experimental volume and magnetic anisotropy of D0_22_ Mn_3_Ga, which has recently become one of the most sought-after materials in order to achieve a stable spin switching at low current density. Despite great progress over the last 10 years, this issue has hitherto remained unsolved. Here, taking into account the effects of strong electronic correlations beyond what is included in standard DFT, we show by comparison with the experiment that the DFT+U method is capable of quantitatively describing the volume and the magnetic anisotropy energy (MAE) in this alloy with physically meaningful choice of onsite Coulomb-U parameter. For the first time using a plane-wave code, we decompose MAE into spin channel-resolved components in order to determine spin-flip and spin-conserving contributions. The Mn atom at the tetrahedral site is identified as the primary source of the high perpendicular MAE with the most dominant spin-orbit coupling (SOC) occurring between its two orbital pairs: ↑↑ coupling and ↓↓ coupling between ${{\boldsymbol{d}}}_{{{\boldsymbol{x}}}^{{\bf{2}}}-{{\boldsymbol{y}}}^{{\bf{2}}}}$ and *d*_*xy*_, and ↑↓ coupling between *d*_*yz*_ and ${{\boldsymbol{d}}}_{{{\boldsymbol{z}}}^{{\bf{2}}}}$. Using the SOC-perturbation theory model, we provide interpretation of our numerical results. These results are important for quantitative microscopic understanding of the large perpendicular MAE observed in this material, and should assist in harnessing its potential for applications in futuristic spintronic devices.

## Introduction

A tetragonal (D0_22_) Heusler alloy Mn_3_Ga has recently created increasing interest among researchers because of its excellent combination of properties, such as low Gilbert damping constant (*α* < 0.008)^1^, small saturation magnetization (M_*s*_ ~ 250 emu/cm^3^)^2^, high Curie temperature (T_C_ > 770 K)^3^, large spin polarization close to that of a half-metal (P ~ 88%)^4^, and strong perpendicular magnetocrystalline anisotropy (K_*u*_ > 10 Merg/cm^3^)^2^. Low Gilbert damping and saturation magnetization but high Curie temperature and spin polarization are necessary preconditions for advanced spintronic applications in order to realize low switching currents and high efficiency of spin injection^5^. High values of perpendicular magnetocrystalline anisotropy (PMA) are preferred to stabilize the perpendicular magnetization against thermal fluctuations, ensuring non-volatility of the stored information particularly when scaling down materials for high density magnetic data storage.

Mn_3_Ga bulk in its tetragonal D0_22_ structure (I4/mmm space group, number 139) has experimental lattice parameters of a = 3.90 Å and c = 7.12 Å^3^. Its structure optimization using density functional theory (DFT) with the Perdew-Burke-Ernzerhof (PBE) functional gives a = 3.78 Å and c = 7.10 Å. These values lead to about 6% smaller lattice volume compared to the experimental one, standing in stark contrast to the well-known trend that the PBE calculations^2^ systematically overestimate the experimental lattice volume. This contrast indicates that a strong Coulomb correlation beyond the traditional DFT is likely in operation. Furthermore, experimental measurements are known to yield the perpendicular (⊥) magnetocrystalline anisotropy energy (MAE) of 14 × 10^6^ erg/cm^3^ (1 meV)^6^. The value theoretically obtained using first-principles DFT overestimates the experimental value by a factor of about 2. This inspires us to investigate the effect of intra-site Coulomb correlation on the magnetic anisotropy of Mn_3_Ga using DFT+U approach (see Method section).

**Figure 1:**
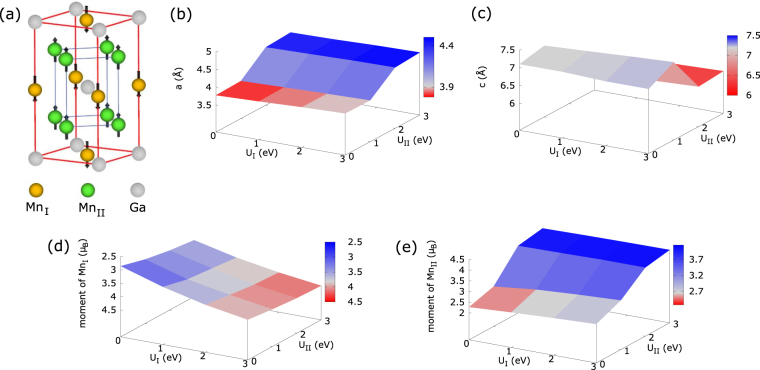
(**a**) Crystal structure of D0_22_ Mn_3_Ga under investigation, and calculated (**b**) in-plane lattice constant a (**c**) out-of-plane lattice constant c (**d**) magnetic moment of Mn_I_ and (**e**) magnetic moment of Mn_II_ as a function of effective U parameter.

In D0_22_ Mn_3_Ga crystal, Mn atoms occupy two different positions [see Fig. 1(a)]. The first position (Mn_I_), with multiplicity 1, is located at the Wyckoff position 2b (0, 0, 0.5) [octahedral site] and the second position (Mn_II_), with multiplicity 2, is at 4d (0, 0.5, 0.25) [tetrahedral site]^7^, indicating that the effective U can be potentially different for these two different sites. A proper choice of the effective U parameter in PBE+U formalism is crucial in understanding and interpreting the results of first-principles calculations. Therefore, we scan the U_I_, U_II_ parameter space [U_I_ = U(Mn_I_), U_II_ = U(Mn_II_)] and obtain the best set of U_I_ and U_II_ values (U_I_ = 2.6 eV and U_II_ = 0 eV), yielding in-plane lattice constant, out-of-plane lattice constant, magnetic moment of Mn_I_ and magnetic moment of Mn_II_ close to the respective experimental ones simultaneously [see Fig. 1(b–e), the light-grey color corresponds to the experimental value in these subfigures].

With U_I_ = 2.6 eV (U_I_ = 0 eV), the optimized in-plane lattice parameter is determined to be 3.9054 Å (3.78 Å), while the out-of-plane lattice parameter is 7.0518 Å (7.10 Å). Both lattice parameters with U_I_ = 2.6 eV are in close agreement with existing experimental lattice parameters of 3.90 Å and 7.12 Å^3^. The optimized structure possesses a ferrimagnetic ordering, with the Mn_I_ atoms aligned antiparallel to the Mn_II_ atoms. For U_I_ = 2.6 eV (U_I_ = 0 eV), the Mn_II_ atoms are separated from their nearest Mn_II_ neighbors by 2.76 Å (2.67 Å) and from the nearest Ga atoms by 2.63 Å (2.59 Å), while the Mn_I_ atoms are 2.63 Å (2.59 Å) from the nearest Mn_II_ atoms and 2.76 Å (2.67 Å) from the nearest Ga atoms. For U_I_ = 2.6 eV (U_I_ = 0 eV), the magnetic moments are −3.895 *μ*_*B*_ (−2.857 *μ*_*B*_) for the Mn_I_ atoms and +2.533 *μ*_*B*_ (+2.315 *μ*_*B*_) for the Mn_II_ atoms, giving the structure an overall magnetic moment of +2.206 *μ*_*B*_ (+1.7165 *μ*_*B*_) per formula unit. The total magnetic moment with U_I_ = 2.6 eV agrees better with existing experimental results of 2.2 *μ*_*B*_ per formula unit^4^.

**Figure 2:**
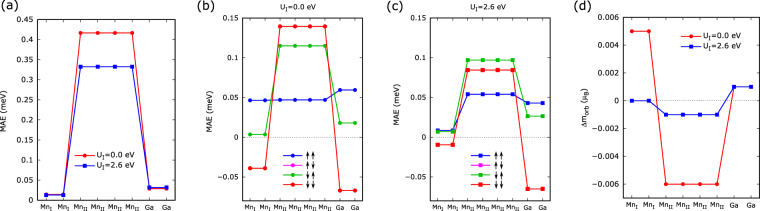
(**a**) Calculated atom-resolved MAE of D0_22_ Mn_3_Ga for U(Mn_I_) = 0.0 and = 2.6 eV; its spin channel- and atom-resolved MAE for (**b**) U(Mn_I_) = 0.0 eV and (**c**) U(Mn_I_) = 2.6 eV; and (**d**) atom-resolved orbital-magnetic-moment anisotropy of D0_22_ Mn_3_Ga for U(Mn_I_) = 0.0 and = 2.6 eV.

With U_I_ = 2.6 eV (U_I_ = 0 eV), the bulk structure exhibits a ⊥ MAE of +1.41 (+1.76) meV. The atom-resolved MAE is shown in Fig. 2(a). The Mn_I_ atoms make a near-zero positive contribution (0.013 meV/atom for U_I_ = 2.6 eV and 0.014 meV/atom for U_I_ = 0 eV) to the MAE. Although adding U(Mn_I_) of 2.6 eV does not show a considerable change in the net MAE of Mn_I_ atom, it changes the spin-channel-resolved MAE significantly, ↑↑ and ↓↓ contributions in particular [compare Fig. 2(b,c)]. The Ga atoms also make a small positive contribution to the MAE, but the Mn_II_ atoms are primary source of the high PMA, with each atom making a large positive contribution (about 0.33 meV/atom for U_I_ = 2.6 eV and 0.42 meV/atom for U_I_ = 0 eV). Figure 2(d) shows the anisotropy of orbital magnetic moment. This anisotropy is smaller for the PBE+U calculation in agreement with the finding that the PBE+U rather than PBE gives a smaller MAE for Mn_3_Ga.

Within the framework of second-order perturbation theory^8^,

1 $${{\rm{MAE}}}^{\sigma \sigma ^{\prime} }={\xi }^{2}\,\sum _{o,u,\sigma ,\sigma ^{\prime} }\,(2{\delta }_{\sigma \sigma ^{\prime} }-1)\,\frac{{|\langle {o}^{\sigma }|{L}_{z}|{u}^{\sigma ^{\prime} }\rangle |}^{2}-{|\langle {o}^{\sigma }|{L}_{x}|{u}^{\sigma ^{\prime} }\rangle |}^{2}}{{\varepsilon }_{u}^{\sigma }-{\varepsilon }_{o}^{\sigma ^{\prime} }},$$

where ${u}^{\sigma }({o}^{\sigma ^{\prime} })$ and ${\varepsilon }_{u}^{\sigma }({\varepsilon }_{o}^{\sigma ^{\prime} })$ respectively stand for eigenstates and eigenvalues of unoccupied (occupied) states in spin state *σ*(*σ*′), *ξ* is the SOC coefficient, and *L*_*z*_ and *L*_*x*_ are the angular momentum operators. Relative contributions of the nonzero matrix elements with the *d*-states are as follows: $\langle {d}_{xz}|{L}_{z}|{d}_{yz}\rangle =1$, $\langle {d}_{{x}^{2}-{y}^{2}}|{L}_{z}|{d}_{xy}\rangle =2$, $\langle {d}_{{z}^{2}}|{L}_{x}|{d}_{yz}\rangle =\sqrt{3}$, $\langle {d}_{{x}^{2}-{y}^{2}}|{L}_{x}|{d}_{yz}\rangle =1$ & $\langle {d}_{xy}|{L}_{x}|{d}_{xz}\rangle =1$. For these nonvanishing matrix elements (two for L_*z*_ and three for L_*x*_ operators), the most dominant contribution to the MAE comes from the states near the Fermi level and its behavior is essentially determined by the denominator of Eq. (1). The SOC interaction between states with the same (different by 1) magnetic quantum number(s), m, is through the L_*z*_ (L_*x*_) operator. For parallel mutual spin orientations (*σσ*′ = ↑↑ or ↓↓), positive (negative) contribution comes from the L_*z*_(L_*x*_) coupling; whereas for antiparallel mutual spin orientations (*σσ*′ = ↑↓ or ↓↑), Eq. (1) has opposite sign and thus positive (negative) contribution comes from the L_*x*_(L_*z*_) coupling.

**Figure 3:**
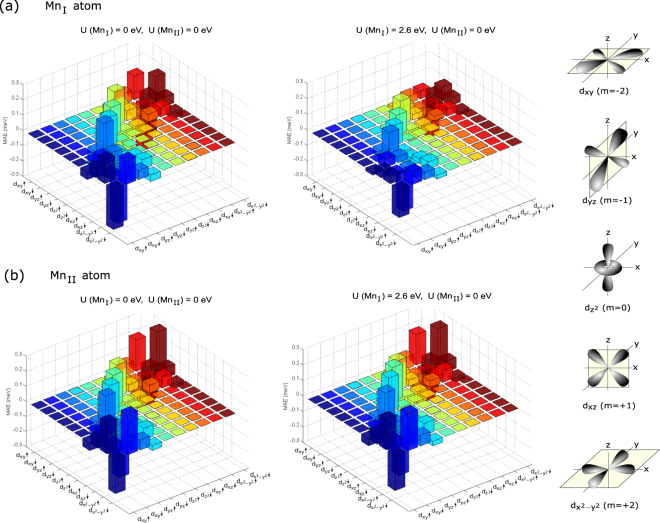
Calculated orbital- and spin channel-resolved MAE for (**a**) Mn_I_ and (**b**) Mn_II_ without (left-panel) and with U(Mn_I_) of 2.6 eV (right-panel).

Figure 3 shows the orbital- and spin-resolved MAE for the (a) Mn_I_ and (b) Mn_II_ atoms. For both (a) and (b), we present results for U(Mn_I_) = 0, U(Mn_II_) = 0 eV (left panel) and U(Mn_I_) = 2.6, U(Mn_II_) = 0 eV (right panel) in order to illustrate the impact of correlations. The left panel of Fig. 3(a) shows that large negative (in-plane) contributions to the MAE come from *σσ*′ = ↑↓ coupling and ↓↑ coupling between the ${d}_{{x}^{2}-{y}^{2}}$ and *d*_*xy*_ orbitals, while relatively small positive (perpendicular or out-of-plane) contributions from ↑↑ coupling and ↓↓ coupling also occur between these orbitals. Additional positive contributions come from ↓↑ coupling and ↑↓ coupling between ${d}_{{x}^{2}-{y}^{2}}$ and *d*_*yz*_ orbitals, ${d}_{{z}^{2}}$ and *d*_*yz*_ orbitals, and d_*xz*_ and d_*xy*_ orbitals, while ↑↓ coupling and ↓↑ coupling between the *d*_*yz*_ and *d*_*xz*_ orbitals gives a small negative contribution. The most notable difference between the orbital-resolved MAE of Mn_II_ and that of Mn_I_ is that the *d*_*xy*_ and ${d}_{{x}^{2}-{y}^{2}}$ orbitals of Mn_II_ atoms contribute to larger PMA due to stronger ↓↓ coupling and ↑↑ coupling, while these orbitals of Mn_I_ atoms give larger in-plane contribution due to stronger ↓↑ coupling and ↑↓ coupling. Like Mn_I_ atoms, in the case of Mn_II_ atoms, the *d*_*yz*_ and ${d}_{{z}^{2}}$ orbitals also make a significant ⊥ contribution through ↑↓ coupling and ↓↑ coupling; while small in-plane contributions come from the ↑↑ coupling and ↓↓ coupling between the d_*xz*_ and *d*_*xy*_ orbitals, and from ↑↓ coupling and ↓↑ coupling between the d_*xz*_ and *d*_*yz*_ orbitals. With applying the Hubbard U correction of 2.6 eV at Mn_I_ site, similar trends are found as above but the magnitudes of the contributions are reduced significantly. For Mn_I_ atom, as the reductions of positive and negative contributions are of opposite sign [see the right panel of Fig. 3(a)], the changes cancel out and the net MAE of Mn_I_ atom does not show a considerable change by the applied U(Mn_I_) of 2.6 eV. The U correction of 2.6 eV at Mn_I_ site also influences the MAE of Mn_II_ site [see the right panel of Fig. 3(b)], leading to a reduction of about 25% of total MAE in Mn_3_Ga.

**Figure 4:**
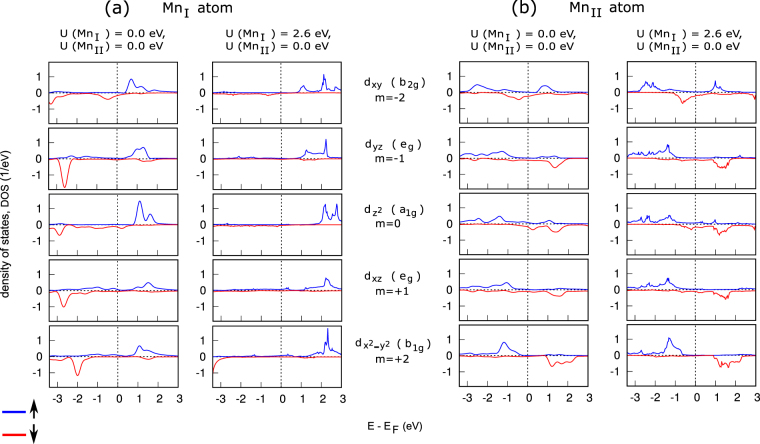
Calculated orbital- and spin- resolved electronic density of states of Mn_3_Ga for (**a**) Mn_I_ and (**b**) Mn_II_ without (left-panel) and with U(Mn_I_) of 2.6 eV (right-panel).

**Figure 5:**
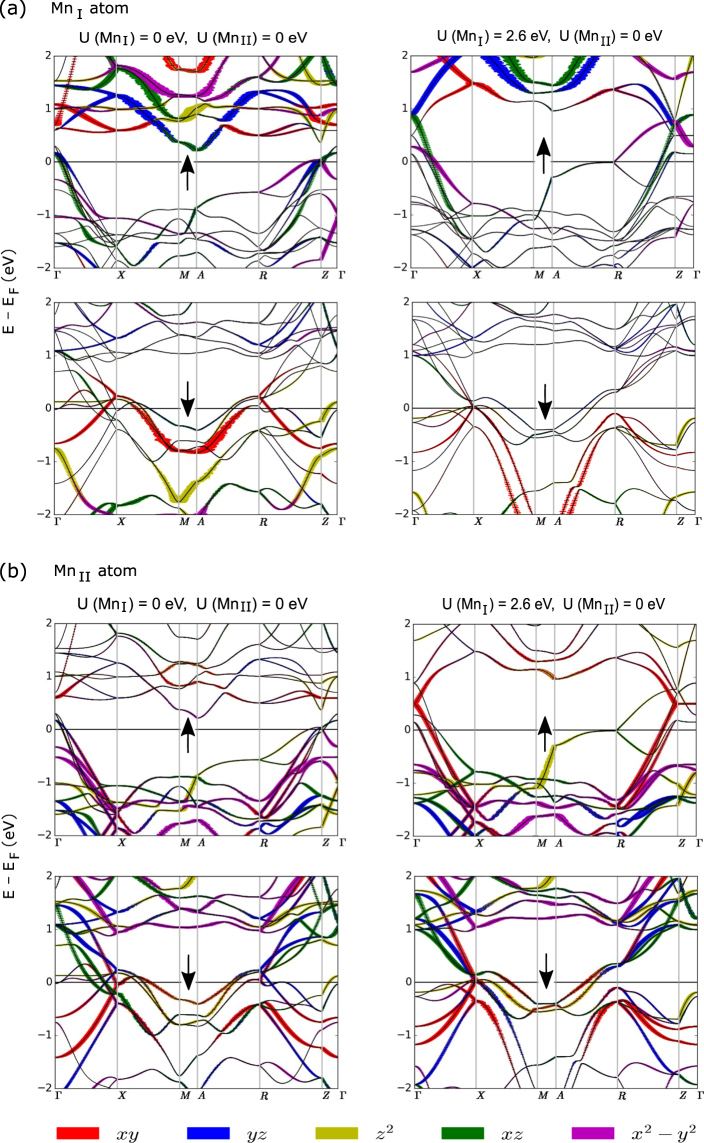
Calculated orbital- and spin- resolved electronic band structure of Mn_3_Ga for (**a**) Mn_I_ and (**b**) Mn_II_ without (left-panel) and with U(Mn_I_) of 2.6 eV (right-panel).

The orbital-projected density of states and band-structure, shown respectively in Figs 4 and 5, provide complementary information about the character of spin-orbit coupling in the (a) Mn_I_ and (b) Mn_II_ atoms. We first discuss our calculated DOS and band-structure in the case of U(Mn_I_) = U(Mn_II_) = 0 eV. The left panel of Fig. 4(a) shows that strong ↑↓ coupling (leading to in-plane MA) occurs between unoccupied ${d}_{{x}^{2}-{y}^{2}}\,\uparrow$ and occupied ${d}_{xy}\,\downarrow$ states, while relatively weak ↑↑ coupling and ↓↓ coupling (leading to PMA) occur between the occupied ${d}_{{x}^{2}-{y}^{2}}\,\uparrow$ and unoccupied ${d}_{xy}\,\uparrow$ states, and between the unoccupied ${d}_{{x}^{2}-{y}^{2}}\,\downarrow$ and occupied ${d}_{xy}\,\downarrow$ states. The left panel of Fig. 5(a) shows that an unoccupied ${d}_{{x}^{2}-{y}^{2}}\,\uparrow$ band exists along the X-M and A-R lines and the existence of an occupied ${d}_{xy}\,\downarrow$ band along these lines suggests that the X-M and A-R lines are the abode of the strong ↑↓ coupling between these orbitals. For the ${d}_{{z}^{2}}$ and *d*_*yz*_ orbital pair, the ↑↓ coupling (PMA) occurs between unoccupied ${d}_{yz}\,\uparrow$ and occupied ${d}_{{z}^{2}}\,\downarrow$ states, mostly near the Γ point on the Γ-X line, near the M point on the X-M line and near the A point on the A-R line. Relatively weak ⊥ contribution comes from the ↑↓ coupling between the unoccupied d_*xz*_ ↑ and occupied *d*_*xy*_ ↓ states. The presence of an unoccupied *d*_*xz*_ ↑ band and occupied *d*_*xy*_ ↓ band along Γ-X-M line suggests this coupling. A weak ⊥ contribution comes from the ↓↑ coupling between the unoccupied d_*yz*_ ↓ and occupied ${d}_{{x}^{2}-{y}^{2}}\,\uparrow$ bands, mostly along the Γ-X and R-Z lines. The ↑↓ coupling also occurs between the unoccupied *d*_*yz*_ ↑ and occupied d_*xz*_ ↓ states mostly around the X point, leading however to a small in-plane contribution. A much stronger presence of the unoccupied ${d}_{{x}^{2}-{y}^{2}}\,\downarrow$ state near the Fermi level in Mn_II_ than in Mn_I_ [see Figs 4(b) and 5(b)] explains why the coupling between ${d}_{{x}^{2}-{y}^{2}}\,\downarrow$ and *d*_*xy*_ ↓ pair gives much larger ⊥ contribution to MAE in Mn_II_ than in Mn_I_. For the *d*_*yz*_ and ${d}_{{z}^{2}}$ pair at Mn_II_ site, the ↑↓ coupling (PMA) occurs between unoccupied *d*_*yz*_ ↑ and occupied ${d}_{{z}^{2}}\,\downarrow$ states, mostly near the Γ point on the Γ-X line. Relatively weak ⊥ contribution comes from the ↑↓ coupling between the unoccupied d_*xz*_ ↑ and occupied *d*_*xy*_ ↓ states. The presence of an unoccupied *d*_*xz*_ ↑ band and occupied *d*_*xy*_ ↓ band along Γ-X-M line suggests this coupling. Similarly weak ⊥ contribution comes from the ↑↓ coupling between the unoccupied d_*yz*_ ↑ and occupied ${d}_{{x}^{2}-{y}^{2}}\,\downarrow$ bands, mostly near the Γ point on the Γ-X line. The ↑↓ coupling also occurs between the unoccupied *d*_*yz*_ ↑ and occupied d_*xz*_ ↓ states mostly around the X point, leading however to a small in-plane contribution.

In the presence of on-site U correction of 2.6 eV at Mn_I_, one feature common to both Mn_I_ and Mn_II_ atoms is that the correction results in a large shift of spectral weight away from the Fermi level. Pushing away the bands near the Fermi level by on-site U causes significant reduction of electron states at and near E_*F*_ which eventually leads to a reduction of about 25% of total MAE in Mn_3_Ga.

In conclusion, taking into account the effects of strong electronic correlations, we show by comparison with the experiment that the DFT+U method is capable of quantitatively describing the volume and the MAE in D0_22_ Mn_3_Ga ferrimagnet. For the first time using a plane-wave code, we decompose MAE into spin channel-resolved components to determine spin-flip and spin-conserving contributions. The Mn atom at the tetrahedral site is identified as the main source of the high ⊥ MAE with the most dominant spin-orbit coupling (SOC) occurring between its two orbital pairs: ↑↑ coupling and ↓↓ coupling between ${d}_{{x}^{2}-{y}^{2}}$ and *d*_*xy*_, and ↑↓ coupling between *d*_*yz*_ and ${d}_{{z}^{2}}$. Using the SOC-perturbation theory model, we provide interpretation of our numerical results. These results are important for quantitative microscopic understanding of the large PMA in this material, and should assist in the development of the futuristic spintronic devices.

## Method

Our calculations are performed using the VASP^9^ implementation of DFT, with the Perdew-Burke-Ernzerhof exchange-correlation functional and projector augmented wave (PAW) potentials^10–14^. Kohn-Sham wave functions are represented using a plane-wave basis truncated at an energy cutoff of 40 Ry. Brillouin zone integrations are done on a uniform Monkhorst-Pack^15^ **k** grid of 19 × 19 × 11. The effect of Coulomb correlation is incorporated using DFT+U approach of Dudarev, in which an effective, rotationally-invariant, screened, onsite Coulomb U (U_*d*_ − J) is added to the DFT functional^16–18^. Atomic positions are fully relaxed using the conjugate gradient algorithm until all inter-atomic forces are smaller than 0.1 meV/Å. The MAE is determined by applying spin-orbit coupling and comparing the total energy values for in-plane and out-of-plane magnetization orientations, according to the following equation: MAE = *E*_100_ − *E*_001_, where (100) and (001) representing the in-plane and out-of-plane orientations, respectively.
